# Gut microbiota dysbiosis in endometriosis: mechanistic insights and gut microbiota-targeted therapeutic strategies

**DOI:** 10.3389/fmicb.2026.1776574

**Published:** 2026-06-05

**Authors:** Lili Liang, Lu Min, Junbo Liu, Ying Liu, Wei Cheng

**Affiliations:** 1 Department of Gynaecology, The Affliated Hospital to Changchun University of Chinese Medicine, Changchun, Jilin, China; 2 Medical Department, The First Clinical Hospital of Jilin Academy of Traditional Chinese Medicine, Changchun, Jilin, China; 3 Changchun University of Chinese Medicine, Changchun, Jilin, China

**Keywords:** endometriosis, fecal microbiota transplantation, gut microbiota, inflammation, microbial translocation

## Abstract

Endometriosis (EMs) is a prevalent, estrogen-dependent gynecological disorder characterized by the ectopic implantation and proliferation of endometrial-like tissue outside the uterine cavity, affecting approximately 10% of reproductive-aged women globally. Despite its high incidence, the exact pathogenesis of EMs remains incompletely elucidated, and current clinical treatments are often limited by suboptimal efficacy and adverse effects. Accumulating evidence over the past decade has revealed a strong observational association between gut microbiota dysbiosis and EMs development, suggesting that the gut microbiota may serve as a novel potential target for understanding and managing this disease. This review systematically summarizes the potential mechanistic links underlying the interplay between gut microbiota dysbiosis and EMs progression, focusing on three core pathways: intestinal barrier dysfunction and microbial translocation, immune dysregulation and ectopic lesion immune escape, and estrogen metabolism disorder mediated by microbial enzymes and metabolites. In addition, this review stratifies gut microbiome profiles by EMs clinical subtypes (peritoneal, ovarian, deep infiltrating), clarifies anatomical correlations of the gut-lesion axis, and discusses confounding factors and causal inference methodologies. Beyond mechanistic insights, this review also discusses emerging gut microbiota-targeted therapeutic strategies for EMs, including probiotic supplementation, prebiotic intervention, fecal microbiota transplantation (FMT), and dietary modulation, with supplementary ethical considerations for FMT. Collectively, this review provides a comprehensive overview of the gut microbiota-EMs axis, highlighting current evidence levels and offering perspectives for the development of innovative, effective, and safe therapeutic approaches for EMs patients.

## 1 Introduction

Endometriosis (EMs) is characterized by the presence of functional endometrial glands and stroma outside the uterine cavity, with four major clinical-pathological subtypes: ovarian, peritoneal, deeply infiltrating, and extrapelvic endometriosis (Maruyama et al., 2020; Wepy et al., 2024). These subtypes exhibit marked heterogeneity in lesion location, invasive capacity, inflammatory microenvironment, and clinical prognosis, suggesting divergent pathophysiological mechanisms and potential subtype-specific microbiome signatures (Wang et al., 2023; Ou et al., 2025). Key clinical symptoms include pain, infertility, and pelvic nodules or masses. EMs affects 10–15% of reproductive-aged women globally (around 190 million cases), and its incidence is on the rise (Villacís et al., 2022; Gobjila et al., 2019). Additionally, 20–50% of infertile women are diagnosed with this disorder (Hanson et al., 2017). Observational and preclinical studies indicate that gut microbiota dysbiosis disrupts the homeostasis of women’s reproductive and endocrine systems, and is associated with multiple female reproductive disorders such as EMs, cervical cancer, and ovarian cancer (Cao et al., 2023; Moustakli et al., 2025). The gut microbiota exerts its systemic effects primarily through three interconnected pathways: microbial translocation, immune modulation, and metabolite production (Balakrishnan et al., 2025; Rooks and Garrett, 2016). For EMs, these pathways converge to influence key processes such as ectopic lesion implantation, inflammation, and estrogen homeostasis.

This review aims to synthesize the current state of knowledge regarding the potential role of gut microbiota dysbiosis in EMs pathogenesis, with a focus on the molecular mechanisms that may link the gut microbiota to ectopic lesion development. We further stratify microbiome profiles across peritoneal, ovarian, and deep infiltrating EMs subtypes, integrate anatomical correlation analyses of the gut-lesion axis, discuss confounding factors (chronic pain, medication use, comorbidities, menstrual cycle) and causal inference methods (including Mendelian randomization). We will also discuss the preclinical and early clinical evidence supporting gut microbiota-targeted interventions, including probiotics, prebiotics, FMT, and dietary modifications, as promising therapeutic approaches for EMs. Ethical issues and safety risks of FMT in EMs are specially supplemented. Finally, we will outline the challenges and future directions in this rapidly evolving field, emphasizing the need for interdisciplinary research to translate preclinical findings into clinical practice. Recent advances in gut organoid-microbiome co-culture and single-cell sequencing are also integrated to update mechanistic evidence. By integrating insights from microbiology, immunology, and gynecology, this review seeks to provide a comprehensive understanding of the gut microbiota-EMs axis and inspire the development of innovative treatments for this common yet understudied disorder.

## 2 Characteristics of gut microbiota dysbiosis in EMs

### 2.1 Alterations in microbiota composition in EMs

**Figure 1:**
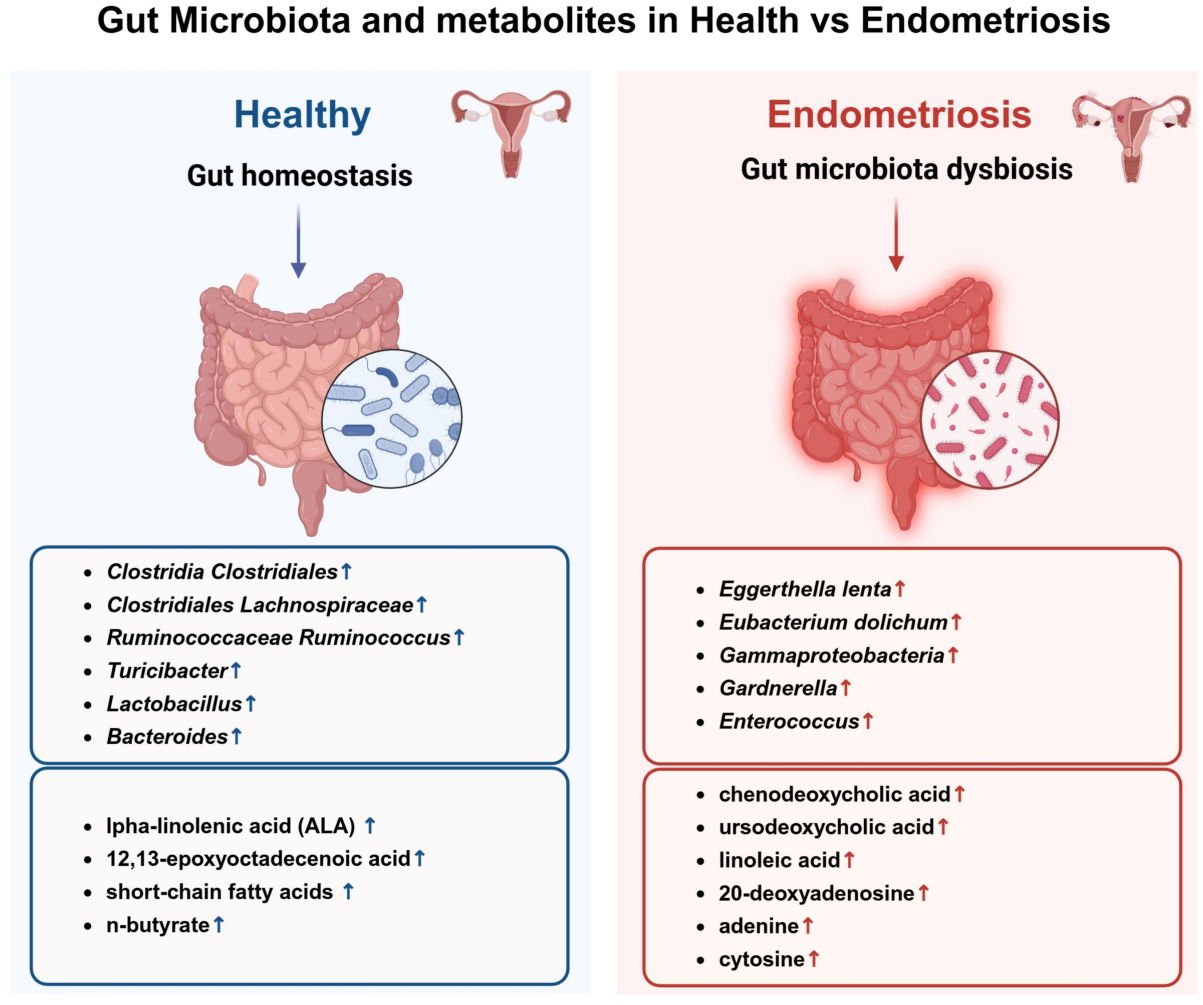
Abnormalities in microbiota and metabolites in EMs.

The intestine constitutes a sophisticated ecosystem, characterized by a homeostatic regulatory loop formed by intestinal mucosal cells, immune cells, and microbial communities that collectively maintain intestinal barrier integrity (Peterson and Artis, 2014; Okumura and Takeda, 2018). Notably, the gut microbiota exerts systemic effects beyond the gastrointestinal tract by modulating the production of pro-inflammatory cytokines and bioactive metabolites (Al Bander et al., 2020; Amoroso et al., 2020). These pro-inflammatory mediators serve as key regulators of extra-intestinal inflammatory processes, with mounting evidence suggesting an association between such gut-derived factors to the pathogenesis of EMs (Machairiotis et al., 2021; Oala et al., 2024). Multiple clinical observational studies have identified distinct gut microbiota compositional changes in EMs patients compared with healthy women. Compared to healthy women, EMs cohorts exhibit elevated abundances of pathogenic taxa including *Gardnerella, Streptococcus, Enterococcus, and Escherichia coli*. Furthermore, fecal microbiota profiling of patients with severe EMs reveals significant alterations in the proportional distribution of *Shigella* and *Escherichia coli*, suggesting a potential correlation between microbiota dysbiosis severity and disease severity rather than establishing causality (Kovács et al., 2021). A previous study conducted 16S rRNA gene sequencing to characterize the gut microbial composition of fecal samples obtained from 12 patients with stage III–IV EMs and 12 demographically matched healthy controls. Their analyses revealed that, relative to the control cohort, the EMs group exhibited diminished gut microbial alpha diversity and an elevated Firmicutes/Bacteroidetes ratio. Moreover, the two groups differed significantly in the abundances of multiple taxa, including *Actinobacteria*, *Enterobacteriaceae*, *Bifidobacterium*, *Dorea*, and *Streptococcus* (Shan et al., 2021). A recent study was designed to explore the association between endometriosis and gut microbiota composition, enrolling 66 women diagnosed with endometriosis and 198 healthy control subjects. Taxonomic analyses revealed that, relative to the control cohort, the endometriosis group exhibited reduced abundances of two genera: one belonging to the class *Bacilli* (*Turicibacter*) and another unclassified genus within the class *Coriobacteriia*. Conversely, an unidentifiable genus in the class *Gammaproteobacteria* was found to be significantly enriched in the endometriosis group (Svensson et al., 2021). In a recent investigation, 41 women were enrolled for fecal microbiota profiling, comprising 20 healthy controls and 21 patients diagnosed with EMs. Comparative taxonomic analyses revealed that *Clostridia Clostridiales, Clostridiales Lachnospiraceae*, and *Ruminococcaceae Ruminococcus* were significantly depleted in the EMs cohort relative to controls. Conversely, two specific taxa *Eggerthella lenta and Eubacterium dolichum* were found to be markedly enriched in patients with EMs (Huang et al., 2021). Collectively, these findings confirm that the gut microbial compositional and proportional profiles diverge markedly between EMs patients and healthy women (Figure 1).

#### 2.1.1 Subtype-stratified gut microbiome signatures in peritoneal, ovarian, and deep infiltrating EMs

Growing evidence demonstrates prominent subtype-specific heterogeneity in gut microbiome profiles among peritoneal, ovarian, and deep infiltrating endometriosis (DIE), reflecting distinct pathophysiological microenvironments and disease progression patterns (Wu et al., 2025; Cai et al., 2025). Peritoneal EMs is closely associated with elevated abundance of Proteobacteria and *Escherichia coli*, along with decreased levels of Lactobacillus and short-chain fatty acid-producing commensal bacteria (Guo and Zhang, 2024). This compositional shift is strongly linked to enhanced peritoneal inflammatory activation and increased microbial translocation into the pelvic cavity, facilitating the early implantation and survival of ectopic endometrial fragments. Ovarian EMs predominantly exhibits enrichment of Bacteroides and Clostridium species, accompanied by significantly elevated *β*-glucuronidase activity and more severe disruption of estrogen metabolism, which aligns with the marked estrogen-dependent growth pattern of ovarian endometriotic cysts and further amplifies local estrogen reabsorption and signaling (Pérez-Prieto et al., 2024). Deep infiltrating EMs (DIE) displays the most severe gut microbial dysbiosis, characterized by markedly reduced alpha diversity, overgrowth of Streptococcus and Enterococcus, and a strong correlation with chronic pelvic pain and intestinal wall invasion (Li et al., 2025). Such microbiome alterations may directly promote nerve invasion, peripheral sensitization, and persistent hyperalgesia, thereby accounting for the intractable pain and high invasiveness typical of DIE. Collectively, these distinct subtype-specific microbial signatures hold great potential as non-invasive differential diagnostic biomarkers and may help predict clinical phenotypes, disease severity, and treatment responses in patients with different EMs variants.

It should be noted that most human studies are cross-sectional and observational. Thus, whether these compositional changes are a cause or consequence of EMs remains unclear. Confounders including diet, menstrual cycle phase, hormonal therapy, prior antibiotic use, chronic pain, NSAID/opioid exposure, and comorbid depression were not fully controlled in these studies, which may affect microbial profiles. Investigations into the potential association between gut microbiota and EMs are also underway in experimental animal models. A prospective randomized experiment was conducted in a murine endometriosis model induced by intraperitoneal endometrial tissue injection. Mice with 42-day persistent endometrial lesions displayed a unique gut microbiota profile. Key discriminative features included an elevated Firmicutes/Bacteroidetes ratio in endometriosis mice, suggesting disease-associated microbial dysbiosis, as well as increased levels of *Bifidobacterium* (Yuan et al., 2018). A recent investigation explored the impact of gut microbiota alteration through antibiotic treatment on EMs progression. The results showed that gut microbial composition undergoes distinct alterations in mice bearing endometriotic lesions. Specifically, the genus *Bacteroides* is markedly enriched in the fecal microbiota of these lesion-bearing animals (Chadchan et al., 2019). Another study demonstrated that at the phylum level, the EM group showed non-significant decreases in *Bacteroidetes* and *Firmicutes* abundances and a marginally higher Firmicutes/Bacteroidetes ratio, whereas *Proteobacteria* and *Verrucomicrobia* abundances were significantly elevated. Among the top 20 genera by abundance, *Allobaculum*, *Akkermansia*, *Parasutterella*, and *Rikenella* were significantly enriched in the EM group, while eight genera (e.g., *Lachnospiraceae*_NK4A136_group, *Lactobacillus*, *Bacteroides*) underwent significant depletion (Ni et al., 2020). Taken together, these studies indicate that distinct microbiota subsets are strongly linked to EMs occurrence and may contribute to the underlying mechanisms of the disease (Figure 1).

### 2.2 Abnormalities in microbiota metabolites in EMs

Although the precise pathogenesis of EMs remains incompletely elucidated, a growing body of evidence supports a potential role of gut microbiota dysbiosis in driving disease progression (Guo and Zhang, 2024; Wang and Wang, 2025). A key mediator of this microbiota-EMs crosstalk is the perturbation of microbiota-derived metabolites-bioactive signaling molecules that regulate systemic inflammation, estrogen metabolic homeostasis, and immune effector responses, all of which constitute core pillars of EMs pathophysiology (Talwar et al., 2022). Unbiased metabolomics and 16S rRNA sequencing were used to define microbiome-metabolome signatures in stool samples from 18 EMs patients and 31 healthy controls. Results confirmed that EMs patients displayed a unique fecal metabolite signature, with 371 distinct metabolites across diverse compound classes initially detected. Statistical filtering further identified 61 metabolites that distinguished the two groups. Among these, 50 metabolites showed reduced abundance and 11 showed elevated levels in EMs patients versus healthy subjects, including linoleic acid, 20-deoxyadenosine, N-formyl-L-methionine, adenine, cytosine, and adenosine (Talwar et al., 2025). Another study found elevated circulating levels of metabolites including 1-eicosatrienoyl-glycerophosphocholine and 1-oleoylglycerophosphocholine were potentially associated with an increased risk of EMs (Yang, 2024). A previous study was designed to elucidate the interplay between fecal metabolomic profiles and gut microbiota composition in EMs model mice. An untargeted metabolomics approach was employed to characterize the fecal metabolite landscape of EMs mice, leading to the identification of 156 annotated differential metabolites. Subsequent analyses revealed that the feces of EMs mice exhibited elevated levels of chenodeoxycholic acid and ursodeoxycholic acid, alongside diminished abundances of alpha-linolenic acid (ALA) and 12,13-epoxyoctadecenoic acid (12,13-EOTrE) (Ni et al., 2020). Another study revealed that perturbed gut microbiota may promote endometriotic lesion progression, with feces from EMs mice containing reduced levels of short-chain fatty acids and n-butyrate relative to non-EMs counterparts (Chadchan et al., 2021). A recent investigation characterized the fecal metabolite landscape in mice with and without EMs. Comparative analyses identified a distinct metabolic signature comprising over 50 differential metabolites in fecal samples from sham-operated mice relative to their EMs-afflicted counterparts. They further visualized the relative abundances of six representative metabolites from this signature: quinic acid, along with cytosine, 1-methyl-histidine, *N*G-dimethyl-L-arginine, 2-aminoheptanoic acid, and *N*-acetylaspartic acid—all of which exhibited divergent abundance patterns in the feces of EMs mice (Chadchan et al., 2023) (Table 1).

**Table 1:** Summary of human studies on gut microbiota and metabolites in EMs.

Study	Design	Sample size (EMs/Control)	Sample type	Key microbial findings	Key metabolite findings	Limitations
Shan et al. (2021)	Cross-sectional	12/12	Fecal	↓α-diversity, ↑*Firmicutes*/*Bacteroidetes*	↑E2, correlated with *Blautia*, *Dorea*	Small sample, stage III–IV only
Svensson et al. (2021)	Cross-sectional	66/198	Fecal	↓*Turicibacter*, ↑unclassified *Gammaproteobacteria*	ND	Limited metabolite detection
Huang et al. (2021)	Cross-sectional	21/20	Fecal	↓*Lachnospiraceae*, ↑*Eggerthella lenta*	ND	Single-center, small sample
Talwar et al. (2025)	Cross-sectional	18/31	Fecal	ND	61 differential metabolites	No paired microbiome analysis

## 3 Mechanistic insights: how gut microbiota dysbiosis drives EMs

### 3.1 Intestinal barrier dysfunction and bacterial translocation

Gut microbiota dysbiosis may disrupt the integrity of the intestinal mucosal barrier, a physical and immunological defense system composed of epithelial cells, tight junction proteins (e.g., occludin, ZO-1), and mucus layers (Stolfi et al., 2022; Barbara et al., 2021). Pathogenic bacteria enriched in EMs, such as *Escherichia coli* and Streptococcus, may secrete pro-inflammatory factors and toxins that degrade tight junction proteins, leading to increased intestinal permeability—a condition known as “leaky gut” (Shu et al., 2023; Wang et al., 2022). This barrier dysfunction may facilitate the translocation of gut microbes or microbial components (e.g., lipopolysaccharide, LPS) and viable bacteria from the intestinal lumen into the systemic circulation (Ghosh et al., 2020; Massier et al., 2021). Circulating LPS can activate the toll-like receptor 4 (TLR4)-nuclear factor kappa-B (NF-κB) signaling pathway in peripheral tissues and ectopic endometrial lesions, triggering a cascade of pro-inflammatory cytokine production (Guo et al., 2023; de Azevedo et al., 2021). These cytokines may promote the proliferation, adhesion, and invasiveness of ectopic endometrial cells, thereby accelerating lesion growth and dissemination (Delbandi et al., 2013). While gut microbiota dysbiosis is associated with EMs, direct causal evidence for gut-peritoneum microbial translocation in human EMs remains limited. Most evidence is from preclinical models. A recent study found that FMT from EMs patients to mice impaired intestinal barrier function, promoting the translocation of gut microbes, particularly *Pseudomonas* into the peritoneal cavity and ectopic lesions. This translocated *Pseudomonas* was confirmed to be a key mediator of LPS-induced NETosis, which may drive EMs pathogenesis (Wu et al., 2025).

#### 3.1.1 Anatomical correlation of the gut-lesion axis

**Figure 2:**
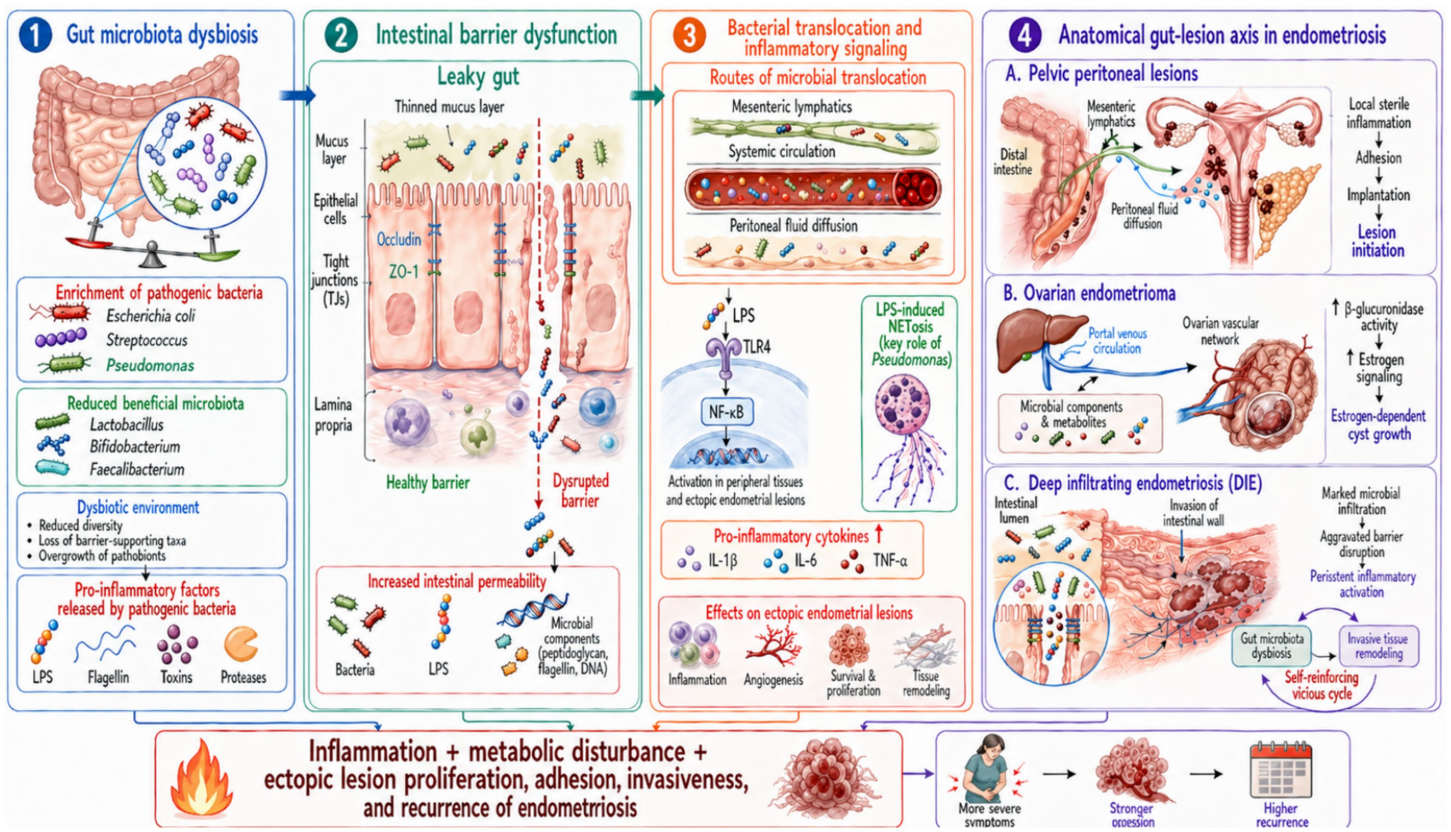
Gut barrier disruption and bacterial translocation in endometriosis. Gut microbiota dysbiosis disrupts the intestinal barrier by reducing mucus protection and tight junction integrity, thereby increasing intestinal permeability. Translocated bacteria and microbial products, especially LPS, activate TLR4/NF-κB signaling and induce pro-inflammatory cytokine release. These inflammatory signals promote ectopic lesion adhesion, implantation, proliferation, angiogenesis, and tissue remodeling. Different lesion sites may receive microbial stimuli through distinct anatomical routes, contributing to lesion heterogeneity, invasiveness, recurrence, and symptom severity in endometriosis.

The anatomical localization of endometriotic lesions directly determines the routes, efficiency, and functional consequences of microbial translocation along the gut-lesion axis, thereby establishing an anatomical gradient that underpins the subtype-specific inflammatory and metabolic disturbances observed in distinct clinical variants (Mora et al., 2025). For pelvic peritoneal lesions, gut-derived microbes and lipopolysaccharide (LPS) primarily translocate from the distal intestine into the pelvic cavity through mesenteric lymphatic vessels and peritoneal fluid diffusion, which rapidly triggers local sterile inflammation and promotes the adhesion and implantation of ectopic endometrial cells (Gaballah et al., 2024). In the case of ovarian endometriomas, translocated microbial components and metabolites reach the ovarian microenvironment mainly through the portal venous circulation and the ovarian vascular network, where they locally enhance *β*-glucuronidase activity and estrogen signaling, thereby forming a microenvironment that amplifies estrogen-dependent cyst growth and progression (Fu et al., 2025). For deep infiltrating endometriosis (DIE) lesions that directly invade the intestinal wall, a direct anatomical connection between the gut lumen and ectopic lesions is established, which markedly accelerates microbial infiltration, aggravates intestinal barrier disruption, and triggers persistent local inflammatory activation; this direct crosstalk forms a self-reinforcing vicious cycle between gut microbiota dysbiosis and invasive tissue remodeling, ultimately accounting for the more severe clinical symptoms, stronger invasiveness, and higher recurrence rate observed in DIE patients (Chapron et al., 2006; Yin et al., 2023). Collectively, this anatomically distinct pattern of microbial translocation provides a mechanistic explanation for the heterogeneous severity of inflammatory and metabolic disturbances across peritoneal, ovarian, and deep infiltrating endometriosis subtypes (Figure 2).

### 3.2 Immune dysregulation and immune escape of ectopic lesions

Immune cells constitute a vital component of the reproductive tract microenvironment, where they secrete copious amounts of cytokines and chemokines to modulate local tissue homeostasis (Dai et al., 2023; Zhou et al., 2022). Accumulating research evidence indicates that macrophages play a pivotal role in EMs pathogenesis (Zheng et al., 2018), with lesions exhibiting elevated macrophage infiltration and a skewed M2/M1 polarization ratio favoring the anti-inflammatory M2 phenotype. Notably, gut microbiota dysbiosis may drive macrophage-mediated immune imbalance in EMs. For instance, one study demonstrated that *Escherichia coli* exerts a suppressive effect on EMs progression by inducing M1 polarization in peritoneal macrophages and activating the interleukin-1 (IL-1) signaling pathway (Yela et al., 2021). In a separate investigation, a recent study found that gut microbiota dysbiosis promotes the production of *β*-glucuronidase, which in turn drives macrophage polarization, enhances the proliferation, invasion, and migration of endometrial stromal cells, and induces macrophage infiltration, ultimately potentially facilitating EMs onset and development (Li et al., 2025).

**Figure 3:**
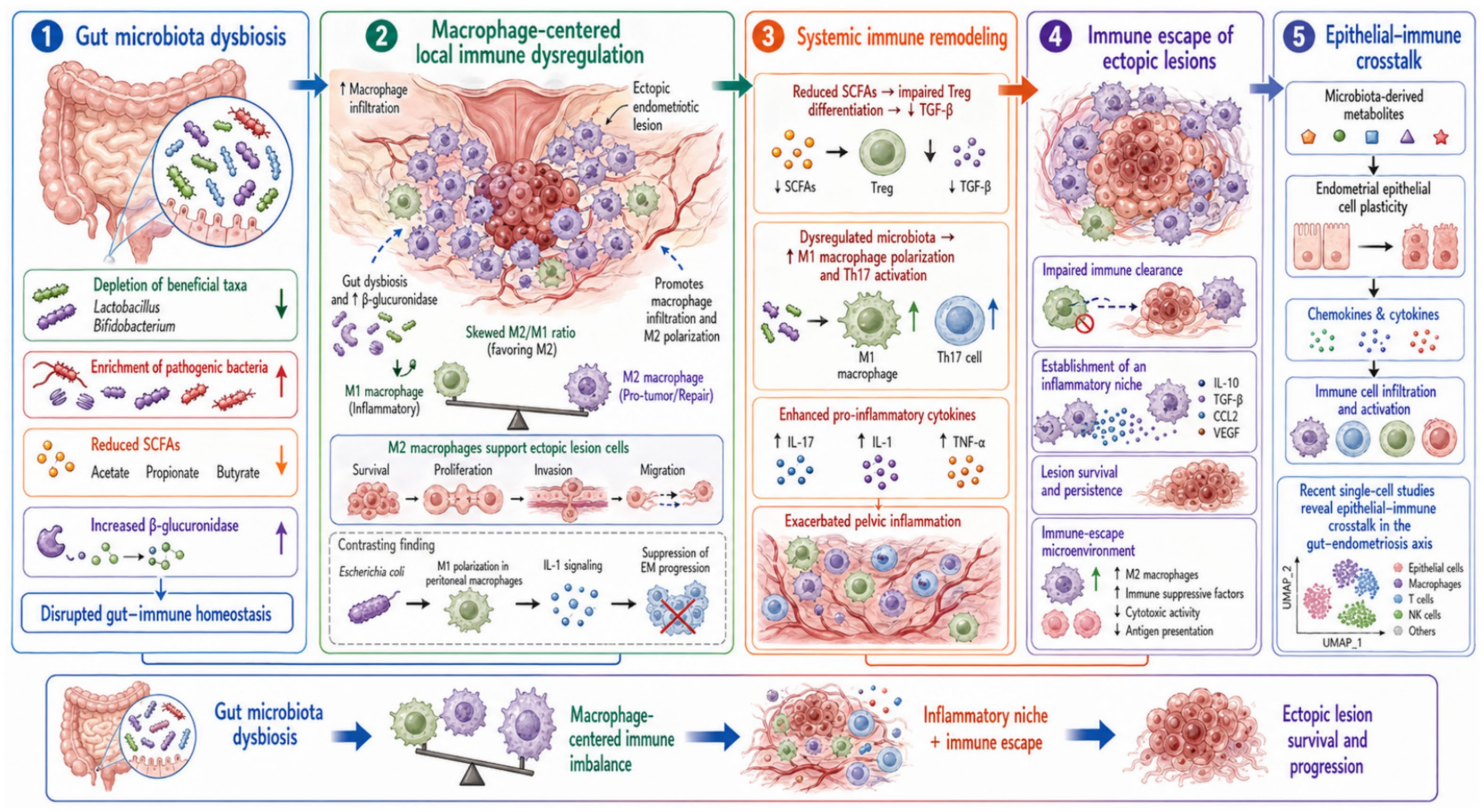
Gut dysbiosis drives macrophage-centered immune imbalance in endometriosis. Gut microbiota dysbiosis reduces beneficial taxa and SCFA production while increasing pathogenic bacteria and β-glucuronidase activity, disrupting gut–immune homeostasis. Dysbiosis promotes macrophage infiltration into ectopic lesions and alters macrophage polarization, with enrichment of anti-inflammatory M2-like macrophages that may support tissue repair-like responses. Meanwhile, reduced SCFAs impair Treg differentiation and favor M1 macrophage polarization, Th17 activation, and pro-inflammatory cytokine release. Together, these immune alterations create a chronic inflammatory yet immune-tolerant microenvironment, thereby supporting the progression of endometriosis.

Gut microbiota dysbiosis further reshapes the systemic immune microenvironment by regulating the differentiation and functional activity of immune cells, thereby contributing to the immune escape of ectopic endometrial lesions (Mora et al., 2025; Tang et al., 2024). Under physiological conditions, commensal microbiota drive the differentiation of regulatory T cells (Tregs) and M2-type macrophages, which collectively maintain immune tolerance and tissue integrity (Pandiyan et al., 2019; Ge et al., 2024). In EMs, however, the depletion of beneficial taxa (e.g., *Lactobacillus* and *Bifidobacterium*) and enrichment of pathogenic bacteria skew the systemic immune response toward a pro-inflammatory phenotype (Datkhayeva et al., 2025). Specifically, reduced levels of short-chain fatty acids (SCFAs)-key metabolites of commensal bacteria-impair Treg differentiation, leading to decreased secretion of immunosuppressive cytokines such as transforming growth factor-*β* (TGF-β) (Ratajczak et al., 2019). Concurrently, dysregulated microbiota promotes the polarization of pro-inflammatory M1 macrophages and the activation of T helper 17 (Th17) cells, exacerbating local inflammation within the pelvic cavity (Lun et al., 2024). This immune imbalance not only compromises the clearance of ectopic lesions but also fosters an inflammatory niche that supports lesion survival and proliferation (Figure 3). Recent single-cell sequencing studies have clarified epithelial-immune crosstalk in the gut-EMs axis, revealing that microbiota-derived metabolites modulate endometrial epithelial cell plasticity and immune cell infiltration at the single-cell level, providing new targets for intervention (Wu et al., 2025; Cao et al., 2026).

### 3.3 Estrogen metabolism disorder: a key amplifying loop

Gut microbiota dysbiosis disrupts estrogen metabolism, creating a positive feedback loop that potentially accelerates EMs progression (Li et al., 2025; Zhang et al., 2025). Estrogen is a key driver of endometrial cell growth, with its bioavailability regulated by the gut-liver axis. Estradiol promotes the adhesion, implantation, and survival of ectopic lesions while inducing the secretion of pro-inflammatory factors (e.g., matrix metalloproteinases, cytokines), and elevated estrogen levels enhance EMs lesion growth and inflammation (Tang et al., 2025).

To elucidate the potential link between gut microbiota and estrogen metabolism in EMs, a cohort study conducted targeted estrogen metabolic profiling in EM patients. Using liquid chromatography–tandem mass spectrometry (LC–MS/MS) to quantify urinary estrogen metabolites and next-generation sequencing (NGS) to analyze 16S rRNA V4 region microbiome data, the researchers identified significant intergroup differences in the levels of 17β-estradiol, 16-keto-17β-estradiol, 2-hydroxyestradiol, and 2-hydroxyestrone between EM patients and healthy controls. Notably, the gut microbial composition of EM patients exhibited a significant positive correlation with urinary estrogen profiles (Le et al., 2021). Another investigation detected elevated concentrations of 4-hydroxyestrone (4OHE1), 2-hydroxyestradiol (2OHE2), and 4-hydroxyestradiol in the eutopic endometrium of EM patients (Othman et al., 2021). Additionally, Shan et al. (2021) observed markedly increased serum estradiol (E2) levels in EM patients, a phenotype that positively correlated with the fecal abundance of *Blautia* and *Dorea* genera.

A core mechanism underlying microbiota-driven estrogen dysregulation involves the microbial enzyme *β*-glucuronidase (Le et al., 2021). Expressed by intestinal bacteria, this enzyme hydrolyzes estrogen-glucuronide conjugates excreted by the liver, releasing free, biologically active estrogen into the systemic circulation-a process termed enterohepatic recirculation (Hu et al., 2023a). Intriguingly, one study found that *β*-glucuronidase expression was upregulated in EM lesions relative to normal endometrial tissue, with the enzyme promoting EM pathogenesis directly or indirectly by impairing macrophage function (Wei et al., 2023). Notably, *β*-glucuronidase and β-glucosidase secreted by intestinal taxa such as *Bacteroides*, *Bifidobacterium*, *Escherichia coli*, and *Lactobacillus* have been reported to facilitate estrogen deconjugation, enhance free estrogen reabsorption, and thereby elevate circulating estrogen levels (Weber et al., 2024). In EMs, the enrichment of β-glucuronidase-producing bacteria (e.g., *Clostridium* species) amplifies this recirculation pathway, leading to systemic estrogen elevation (Hu et al., 2023a).

**Figure 4:**
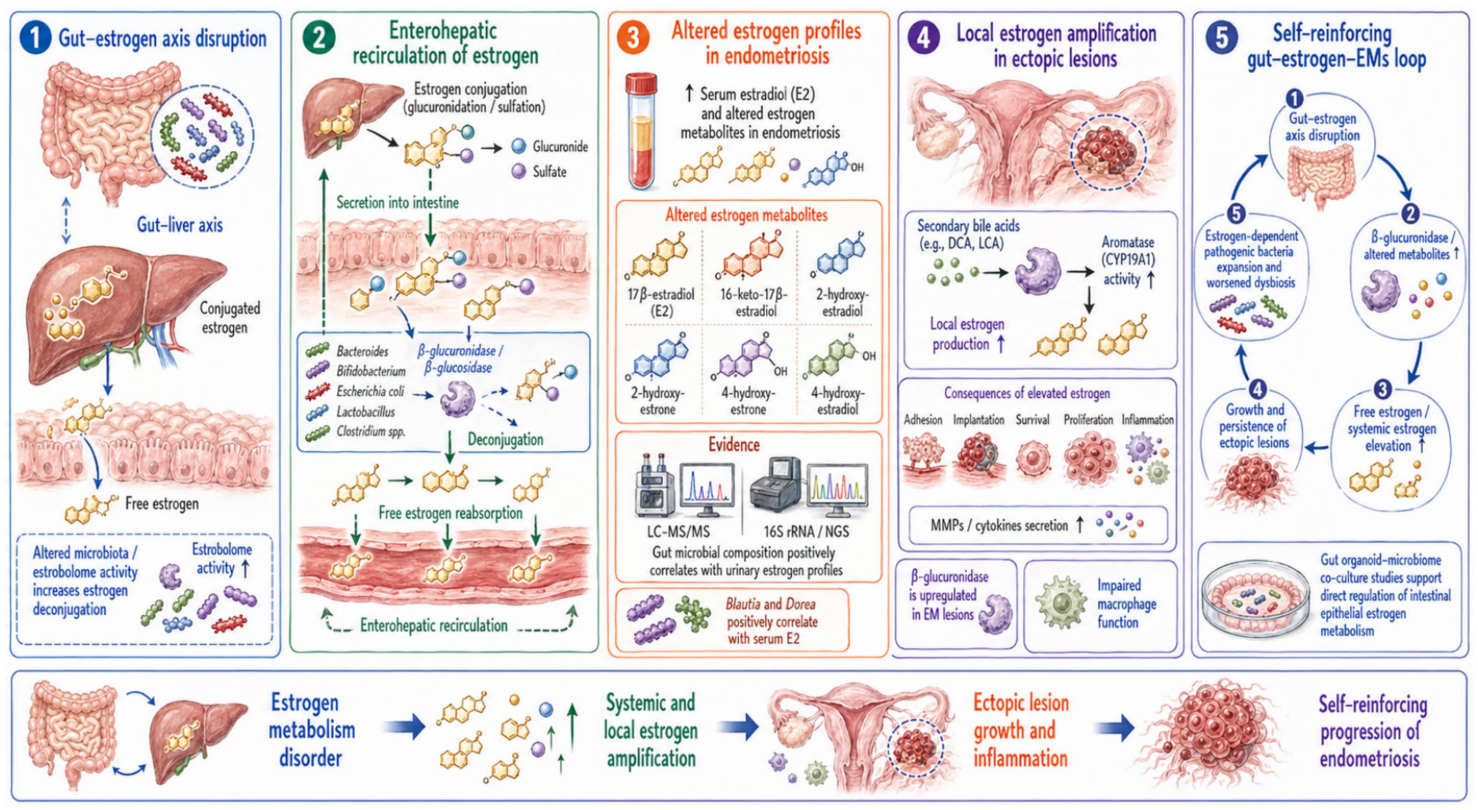
Estrogen dysregulation amplifies endometriosis progression. Gut–estrogen axis disruption alters estrogen metabolism through the gut–liver circulation. Microbial β-glucuronidase and β-glucosidase promote estrogen deconjugation, increasing free estrogen reabsorption and systemic estrogen levels. Altered estrogen profiles and microbiota-derived metabolites further enhance local aromatase activity, leading to estrogen amplification within ectopic lesions. Elevated estrogen promotes lesion adhesion, implantation, survival, proliferation, inflammation, and MMP/cytokine secretion. This bidirectional gut–estrogen–lesion crosstalk forms a self-reinforcing loop that supports endometriosis progression.

Beyond direct enzymatic effects, microbiota-derived metabolites further perturb estrogen homeostasis (Zhang et al., 2025). For instance, secondary bile acids modulate the activity of aromatase—the enzyme responsible for converting androgens to estrogens within ectopic lesions—thereby exacerbating local estrogen production (Phelps et al., 2019; Liu et al., 2018). Critically, elevated estrogen levels not only stimulate the proliferation of ectopic endometrial cells but also foster the growth of estrogen-dependent pathogenic bacteria, which in turn exacerbates gut microbiota dysbiosis (Kumari et al., 2024). This bidirectional crosstalk between gut microbiota and estrogen metabolism constitutes a self-reinforcing cycle that sustains and amplifies the pathogenic cascade of EMs (Table 2, Figure 4). Gut organoid-microbiome co-culture systems in the past two years have verified the direct regulatory effect of key EMs-associated bacteria on intestinal epithelial estrogen metabolism, providing *in vitro* functional evidence for the gut-estrogen-EMs axis (Gnecco et al., 2023; Zhang et al., 2024).

**Table 2:** Mechanistic pathways linking gut microbiota dysbiosis to EMs.

Pathway	Microbial factors	Host pathways	Biological effects	Evidence level
Intestinal barrier dysfunction	Pathogenic bacteria, LPS	TLR4/NF-κB	Inflammation, lesion growth	Preclinical strong; clinical limited
Immune dysregulation	↓SCFAs, ↓*Lactobacillus*	Treg/Th17, M1/M2 imbalance	Immune escape, chronic inflammation	Preclinical strong
Estrogen metabolism disorder	β-glucuronidase-producing bacteria	Enterohepatic recirculation	↑E2, lesion proliferation	Clinical association; preclinical causal

## 4 Gut microbiota-targeted therapeutic strategies for EMs

Accumulating evidence indicates that gut microbiota dysbiosis—marked by reduced beneficial bacteria, enriched pathogenic taxa, and abnormal metabolite profiles—plays a pivotal role in EMs pathogenesis via intestinal barrier dysfunction, immune dysregulation, and estrogen metabolism disorder (Kobayashi, 2023). Consequently, gut microbiota-targeted interventions have emerged as promising preclinical and exploratory therapeutic paradigm for EMs.

### 4.1 Probiotics

Modulating gut microbiota composition and function through probiotics is the most well-studied and accessible strategy for EMs (Kobayashi, 2023). These interventions target the restoration of microbial homeostasis, thereby reversing the pathological cascades induced by dysbiosis. *Lactobacillus acidophilus*, a commensal probiotic bacterium in the human gut, exhibits robust efficacy in mitigating various inflammatory disorders. Emerging evidence highlights its potential to regulate cytokine production by peripheral blood monocytes in patients with EMs (Sari et al., 2022). Itoh et al. (2011a, b) conducted a randomized, double-blind, placebo-controlled trial, demonstrating that oral supplementation with *Lactobacillus gasseri* OLL2809 significantly alleviated dysmenorrhea in patients with EMs. Complementary animal studies further elucidated the underlying mechanism: oral administration of this strain effectively suppressed the growth of ectopic endometrial lesions in mice by elevating peritoneal cavity interleukin-12 (IL-12) levels and enhancing the cytotoxic activity of natural killer (NK) cells. Most probiotic evidence is limited to *Lactobacillus* strains and is either preclinical or from small-scale trials; efficacy is strain-specific, and no probiotic is currently approved for routine EMs treatment. Another study sought to assess the efficacy of *Lactobacillus gasseri* OLL2809 (OLL2809) in treating established abdominal wall endometriotic implants in diestrus Wistar-Imamichi female rats. The results highlight the dual potential of OLL2809, as it is effective not only in managing pre-existing endometriosis but also in inhibiting the growth of endometrial tissue to prevent disease progression (Uchida and Kobayashi, 2013). Given the high interindividual heterogeneity of gut microbiota and the multifaceted pathogenesis of EMs, broader strain screening and large-scale RCTs are needed before clinical application.

### 4.2 Fecal microbiota transplantation (FMT)

FMT refers to the transfer of fecal microbial suspensions from healthy, rigorously screened donors to recipients, with the core objective of restoring the homeostasis of the recipient’s gut microbial ecosystem (Woodworth et al., 2017; Yadegar et al., 2024). This strategy has demonstrated therapeutic efficacy in ameliorating a broad spectrum of diseases. In recent years, FMT has been increasingly translated into clinical practice for managing diverse systemic disorders, including multiple sclerosis, autism spectrum disorder, and obesity (Zikou et al., 2024; Biazzo and Deidda, 2022). Its therapeutic principle hinges on replenishing and reactivating the functional repertoire of beneficial gut microbiota, thereby reversing microbiota dysbiosis and mitigating disease-associated pathological cascades (Zhanel et al., 2023). To elucidate the regulatory role of gut microbiota in EMs, a recent study established an EMs mouse model and subjected these animals to FMT using microbial inocula derived from healthy donors or EMs patients. Subsequent analyses revealed that, relative to untreated model mice, FMT with healthy donor microbiota led to a marked reduction in both the volume and weight of ectopic lesions. In contrast, FMT using samples from EMs patients exacerbated disease progression. Mechanistic analyses demonstrated that healthy donor-derived FMT alleviated EMs through gut microbiota remodeling. It enhanced *α*-diversity, boosted Lactobacillus abundance, and suppressed Bacteroidetes, while simultaneously elevating acetate levels in feces and ectopic lesions. These changes activated the JAK1/STAT3 pathway in lesions, thereby driving macrophage polarization toward the M1 phenotype (Xu et al., 2025). FMT for EMs remains strictly preclinical; no large-scale clinical trials support its safety or efficacy in humans. Standardization, donor screening, long-term safety, and ethical concerns are major unresolved challenges. Ethical considerations for FMT in EMs include: (1) Strict donor screening for infectious diseases, autoimmune disorders, and hormonal abnormalities to avoid pathogen transmission; (2) Risk of post-transplant immunostimulation or immune exhaustion due to repeated infusions; (3) Potential disruption of endocrine balance in reproductive-aged women; (4) Informed consent and psychological stress associated with FMT procedures. These ethical and safety issues must be fully addressed before clinical translation.

### 4.3 Dietary intervention and lifestyle modification

Dietary modulation represents a pivotal, non-invasive strategy for reshaping gut microbial communities. Mounting evidence indicates that dietary patterns and specific nutrients can regulate the core pathophysiological cascades underpinning EMs, including chronic inflammation, estrogen signaling pathways, and microbe-metabolite cross-talk (Meneghetti et al., 2024; Fjerbæk and Knudsen, 2007). The growing interest in dietary and nutritional interventions for EMs management is largely fueled by patients’ demand for accessible, low-risk adjunctive treatment options (Armour et al., 2021; Armour et al., 2019). Most dietary evidence is from preclinical models or small observational studies. Clinical trial evidence remains limited. Preclinical studies in animal models have demonstrated that a low-fiber, amino acid-based diet reduces gut microbial diversity, enriches EMs-associated taxa (e.g., *Prevotella*), and depletes bacteria involved in fermentation pathways. Notably, this dietary pattern also remodels the brain microenvironment, thereby mitigating systemic inflammatory responses (Mancilla et al., 2023). Additionally, several bioactive dietary components exert multi-targeted benefits against EMs: vitamin D, polyunsaturated fatty acids (including omega-3 and omega-6 variants), resveratrol (abundant in grapes and *Polygonum cuspidatum*), and N-acetylcysteine (found in wheat germ, broccoli, onions, and garlic) all exhibit anti-inflammatory, anti-angiogenic, and pro-apoptotic properties. These effects collectively attenuate cell proliferation and oxidative stress in EMs ectopic lesions to varying degrees (Porpora et al., 2013; Sienko et al., 2024; Arablou et al., 2021). Furthermore, nucleotide supplementation promotes the enrichment of beneficial microbes associated with neural development, digestion, and intestinal absorption—such as *Roseburia* and *Akkermansia*—thus preventing gut microbial dysbiosis (Qu et al., 2023). Meanwhile, consumption of fermented wheat germ modulates the gut microbiota structure in rats, reshaping microbial homeostasis. Through the gut-brain axis, this intervention enhances neurotransmitter levels, restores amino acid metabolic function in brain tissue, alleviates neuroinflammation and central pain sensitization, and ultimately mitigates EMs-associated pain (Hu et al., 2023b). A study sought to assess the effects of chamomile and flaxseed on pelvic pain, dyspareunia, and dysmenorrhea in EMs patients. A randomized controlled trial was conducted with 102 participants diagnosed with the condition, and results confirmed that these two interventions yielded notable efficacy in relieving the core pain-related symptoms associated with EMs (Khalajinia et al., 2024). A previous study investigated how a Western diet affects endometriotic lesion development in mice and to identify the associated mechanisms. Results revealed that a Western diet markedly aggravated lesion size in EMs model mice, accompanied by distinct metabolic and immune dysregulations. Specifically, mice with the largest lesions showed a notable loss of the gut bacterium *Akkermansia muciniphila*. Additionally, endometriosis onset triggered significant shifts in intestinal microbiota composition, highlighting a plausible link between diet, intestinal homeostasis, and EMs progression (Parpex et al., 2024).

## 5 Challenges and future directions

### 5.1 Current research challenges

Although extensive research has been conducted on the association between gut microbiota and EMs, several critical limitations persist in this field (Jiang et al., 2021). First, most studies merely establish a correlational link between gut microbiota and EMs, with insufficient mechanistic investigations to delineate the underlying pathways. Notably, large-scale longitudinal cohort studies are lacking to clarify the causal directionality—specifically, whether gut microbiota dysbiosis constitutes a cause or a consequence of endometriosis. Second, the lack of Mendelian randomization (MR) research limits causal inference. MR studies using genetic instrumental variables are needed to verify causal relationships between gut microbiota and EMs, avoiding confounding from reverse causation and environmental factors. Third, most studies do not control for critical confounding factors: chronic pain, NSAID/opioid use, comorbid depression, dietary changes, and menstrual cycle phase all independently alter gut microbiota; hormonal fluctuations across the cycle lead to dynamic microbiota changes that are rarely considered in sampling timing. Fourth, inconsistencies in sample types (fecal versus mucosal specimens), detection platforms, and analytical methodologies across studies severely compromise the comparability and reproducibility of findings, compounded by the scarcity of large-scale, multicenter investigations. Fifth, the high interindividual variability in gut microbiota composition, coupled with unclear optimal intervention timing and duration, has hindered the translation of preclinical insights into clinical practice. Finally, confounders including diet, antibiotic use, hormonal therapy, and menstrual cycle phase are rarely fully controlled. Furthermore, the dearth of large-scale phase III clinical trials underscores the urgent need to address personalized microbiota-targeted strategies as a core priority for future research.

### 5.2 Future research priorities

Future research should focus on near-term, clinically relevant priorities rather than highly speculative technologies. (1) Longitudinal cohort studies: Conduct large, long-term observational studies to clarify causal directionality and control for confounders including diet, antibiotics, and menstrual cycle phase. (2) Standardized pipelines: Establish unified methods for sample collection, storage, 16S rRNA sequencing, and metabolomic analysis to improve comparability across studies. (3) Biomarker validation: Identify and validate non-invasive microbial or metabolite biomarkers in feces, blood, or urine for early EMs screening and severity evaluation, with subtype-specific signatures for peritoneal, ovarian, and DIE variants. (4) Clinical trials: Perform multicenter, randomized, controlled trials to evaluate the safety and efficacy of probiotics, synbiotics, and dietary interventions in EMs patients with standardized outcome measures. (5) Causal inference: Carry out Mendelian randomization studies to confirm causal relationships between gut microbiota and EMs. (6) Preclinical models: Apply gut organoid-microbiome co-culture and single-cell sequencing to refine mechanistic research. (7) FMT optimization: Establish standardized donor screening, ethical norms, and safety monitoring systems for EMs-targeted FMT.

## 6 Conclusion

Gut microbiota dysbiosis is associated with the occurrence and development of EMs, and may exert regulatory effects through multiple potential mechanisms including intestinal barrier dysfunction, immune dysregulation, and estrogen metabolism disorder. Subtype-specific microbiome signatures and anatomical gut-lesion axis correlations further refine the mechanistic network, while chronic pain, medication use, menstrual cycle, and comorbidities are key confounders requiring rigorous control; Mendelian randomization is needed to validate causality. Gut microbiota-targeted strategies (probiotics, FMT, dietary intervention) show promising preclinical and early clinical therapeutic potential, but FMT involves important ethical and safety issues including donor screening, immune adverse events, and pathogen transmission risks. Current research still faces challenges in causality verification and clinical transformation. Most evidence remains observational or preclinical. Routine clinical application is not yet warranted. Future studies should focus on longitudinal cohorts, standardized methods, clinical validation, subtype stratification, causal inference, and updated technologies (gut organoid, single-cell sequencing) to provide novel diagnostic and therapeutic approaches for EMs.
